# Characterization of the Pepper Virome in Oklahoma Reveals Emerging RNA and DNA Viruses

**DOI:** 10.3390/pathogens14101035

**Published:** 2025-10-13

**Authors:** Caleb Paslay, Akhtar Ali

**Affiliations:** Department of Biological Science, Oxley College of Health & Natural Sciences, The University of Tulsa, Tulsa, OK 74104, USA

**Keywords:** emerging pepper viruses, high throughput sequencing (HTS), polymerase chain reaction (PCR), virus detection, virus prevalence

## Abstract

Pepper (*Capsicum* spp.) is an economically valuable crop worldwide including in the United States due to its nutritional benefits in human health and widespread use as a spice or vegetable. Although numerous viruses have been reported infecting peppers in the USA, little is known about the diversity and distribution of pepper-infecting viruses in Oklahoma. To address this knowledge gap, we conducted a comprehensive pepper virome study to identify viruses infecting pepper and their incidence across six different counties in Oklahoma. A total of 310 plant samples including pepper and other potential hosts were collected during the 2021 and 2022 growing seasons. Samples were analyzed using high-throughput sequencing (HTS) and/or reverse transcription-polymerase chain reaction (RT-PCR) assays. Viral contigs identified via HTS were further validated through RT-PCR or PCR assays followed by Sanger sequencing. In total, 17 distinct viruses were detected, including 15 RNA and two DNA viruses, with several representing putatively novel findings. The most prevalent virus was beet curly top virus (BCTV), followed by tomato yellow leaf curl virus (TYLCV), potato yellow dwarf virus/constricta yellow dwarf virus (PYDV/CYDV), and pepper mild mottle virus (PMMoV). Virus incidence varied by season and location, with some surveys showing infection rates exceeding 80%. This study provides the first in-depth characterization of the pepper virome in Oklahoma and valuable insights into the prevalence and distribution of pepper-infecting viruses. These findings will support the development of informed, targeted strategies for virus detection and management in pepper production systems.

## 1. Introduction

Peppers (*Capsicum* spp.) are a diverse group of crops within the plant family Solanaceae and are economically valuable throughout the United States of America (USA) and around the world. Plant viruses have had a significant impact on the production of pepper crops globally [1,2,3]. Previously, ~68 plant viruses were known to infect pepper [4]. However, the number of viruses in pepper has increased and is estimated to comprise >165 viruses [5]. This increase is partly due to the continual advances in novel characterization methods such as high throughput sequencing (HTS) [6,7,8]. To better understand the diversity and presence of plant viruses, plant virologists have implemented HTS technology for the last several years [9,10]. This approach has been considered a virus metagenomic approach, or a virome study. Previous virome studies associated with the *Capsicum* genus have revealed diverse communities of viral pathogens [11,12,13,14].

Viruses infecting pepper within the USA include, but are not limited to; beet western yellows virus (BWYV), bell pepper alphaendornavirus (BPEV), and pepper golden mosaic virus (PepGMV) in Arizona [15,16,17]; alfalfa mosaic virus (AMV), cucumber mosaic virus (CMV), pepper mottle virus (PeMV), potato virus X (PVX), potato virus Y (PVY), tobacco etch virus (TEV), tobacco mosaic virus (TMV), tobacco rattle virus (TRV) in California [18,19], groundnut ringspot virus (GRSV) in Florida [20]; tomato spotted wilt virus (TSWV) in Georgia [21]; belladonna mottle virus (BMV) in Kansas and Iowa [22]; tobacco mild green mosaic virus (TMGMV) in Louisiana [23]; beet curly top virus (BCTV) in New Mexico [24]; tomato mosaic virus (ToMV), tomato ringspot virus (ToRSV), pepper cryptic viruses in Tennessee [13]; pepper huasteco yellow vein virus (PHYVV), pepper vein yellows virus (PeVYV), and tobacco ringspot virus (TRSV) in Texas [25,26,27].

In Oklahoma, previous reports of virus infection in pepper include alfalfa mosaic virus (AMV) [28] and pepper mild mottle virus (PMMoV) [29]; however, extensive surveys have not been conducted to date. The goal of this effort was to conduct a comprehensive pepper virome study in Oklahoma and to determine the presence and diversity of viruses associated with pepper. In addition, weeds and other non-pepper plants located within or adjacent to pepper fields were sampled to identify alternate hosts and gain a broader understanding of virus reservoirs in the surrounding environment. The findings from this study aim to support local growers and researchers by providing data essential for the development of more effective and targeted virus management strategies.

## 2. Materials and Methods

### 2.1. Field Survey and Collection of Samples

**Figure 1:**
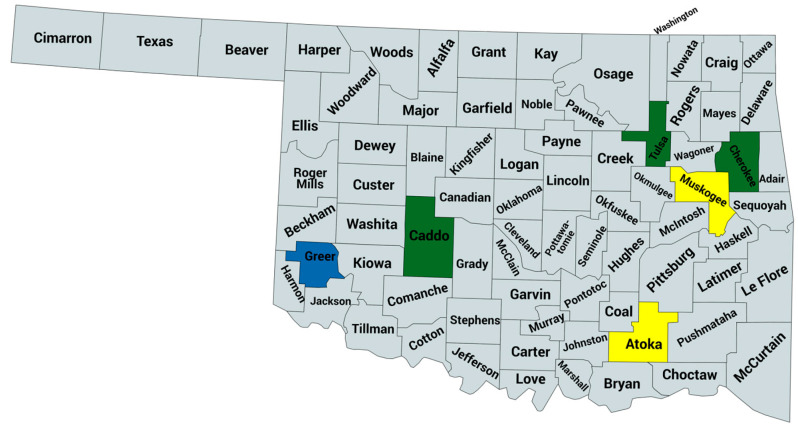
Map of Oklahoma showing counties surveyed during the 2021 and 2022 growing seasons. Counties shaded in green were surveyed in both years, those in yellow were surveyed only in 2021, and those in blue were surveyed in 2022 only. (Created with MapChart).

Field surveys were conducted across six different counties in Oklahoma (Atoka, Caddo, Cherokee, Greer, Muskogee, and Tulsa) during the 2021 and 2022 growing seasons (May to November), targeting regions with active pepper productions (Figure 1). A total of 30 independent surveys were carried out across 12 distinct field sites over the two-year period. A total of 310 leaf samples were collected from both symptomatic and asymptomatic plants, including pepper, weeds, and other crops located within or adjacent to pepper fields. Plant hosts collected include pepper (n = 226), tomato (n = 33), squash (n = 14), potato (n = 5), blackberry (n = 3), basil (n = 2), Johnson grass (*Sorghum halepense*) (n = 2), and a variety of other hosts including pokeweed (*Phytolacca americana*), ivy, peas, white clover (*Trifolium repens*), curled dock (*Rumex crispus*), and bush vetch (*Vicia sepium*) that neighbored pepper fields. Following collections, all samples were transported on ice to the University of Tulsa for further processing.

### 2.2. Nucleic Acid Extraction

Total RNA and DNA were extracted from all collected samples individually using either TRI-reagent^®^ method (Molecular Research Center, Inc., Cincinnati, OH, USA), Spectrum™ Plant Total RNA (Sigma-Aldrich, St. Louis, MO, USA), or E.Z.N.A Plant DNA kit (Omega, Bio-tek, Norcross, GA, USA) according to the manufacturer’s protocols. Isolated nucleic acids were evaluated according to the 260/280 ratios, and the concentration (ng/uL) was quantified using a Nanodrop 8000 (ThermoFisher Scientific, Waltham, MA, USA) spectrophotometer. High quality RNA and DNA were used for further analysis.

### 2.3. Samples Analyzed by HTS, Sample Preparations, and Genome Assemblies

Sixty-three samples of the 310 total samples (20%) were processed by high throughput sequencing (HTS) using a total RNA sequencing approach. Samples processed by HTS were selected based upon distinct virus-like symptoms. Following nucleic acid extraction, total RNA was ribo-depleted using a Plant Ribo-Zero kit (Illumina, San Diego, CA, USA). A cDNA library was prepared for each sample (read size: 75 bp), and sequencing was initiated using the NextSeq 500/550 High-Output v2.5 (Illumina, San Diego, CA, USA) at Oklahoma State University (OSU). Trimmed paired-end reads were cleaned of adaptor sequences and de novo assembled using CLC genomic workbench v.12 (Qiagen, Hilden, Germany) with default parameters (automatic word size, automatic bubble size, minimum contig length = 200, perform scaffolding, mapping reads back to contigs). Assembled contigs sequences were analyzed using BLASTn and BLASTx (NCBI, Bethesda, MD, USA) against the NCBI GenBank databases (nr) for the detection of homologous plant virus sequences. Assembled viral contigs were considered reliable based on the following parameters: average coverage, total read counts, contig length, and percent identity to available sequences on NCBI. A strict cutoff was not applied, as some contigs exhibited lower average coverage but high total read counts. Overall, contigs with higher average coverage and greater total read counts were considered more reliable.

### 2.4. Development of RT-PCR and PCR Assays

Virus specific contigs obtained through HTS were aligned using the ClustalX v2.1 algorithm with publicly available sequences downloaded from the NCBI database (Accessed 19 November 2021, 7 March 2022, 3 June 2022, and 13 September 2022) [30]. Primers targeting specific viruses were designed based on conserved regions shared across various virus isolates of the species in question or were adopted from previously published studies (Supplementary Table S1).

Samples containing contigs representing RNA viruses (n = 5) as identified by HTS, were confirmed using specific RT-PCR assays. Similarly, PCR assays for DNA viruses (n = 2) were designed with specific primers (Supplementary Table S1). The resulting PCR products were visualized on a 1% agarose gel, and either gel-purified or directly purified using a Gel Extraction Kit (Omega, Bio-tek, Norcross, GA, USA) or PCR Purification Kit (Omega, Bio-tek, Norcross, GA, USA). Purified products were Sanger sequenced (Eurofins Genomics, Louisville, KY, USA) for confirmation. The obtained sequences were aligned against the NCBI GenBank database (nr) to validate the presence and identity of the viruses initially detected by HTS. This workflow provided multiple methods for virus confirmation. Furthermore, the developed RT-PCR and PCR assays were applied to all 310 collected samples to screen for the respective RNA and DNA viruses identified by HTS.

### 2.5. Virus Occurrence and Detection Rates

Virus occurrence was calculated as the total number of positive samples for a given virus detected during a field survey, within a county, across a growing season, or in the entire collection. The detection rate was determined by dividing the virus occurrence by the total number of samples collected for the corresponding survey, county, season, or collection. Both HTS and PCR assay results were combined to calculate these values, providing the total number of times each virus was detected.

## 3. Results

### 3.1. Samples and Field Symptoms Associated with Viral Infection

A total of 310 samples were collected from twelve agricultural fields across six counties (Table 1), with the majority consisting of pepper (n = 226 or 73%). Additional host plants included tomato (*Solanum* spp.), potato (*Solanum* spp.), squash (*Cucurbita* spp.), blackberry (*Rubus* spp.), and Johnson grass (*Sorghum* spp.). The largest number of samples came from Caddo County (n = 124) in both years due to a greater acreage of pepper fields (>600 acres). Pepper was sampled most frequently due to its primary importance, while tomato (n = 33) was also commonly collected because of its physical proximity and taxonomic relationship to pepper within the family Solanaceae.

**Figure 2:**
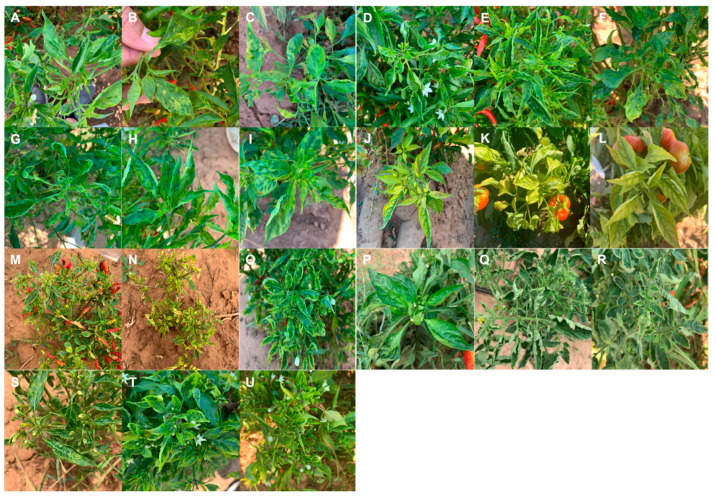
Representative symptoms observed on pepper and tomato plants infected with various viruses detected via high-throughput sequencing (HTS) and PCR. Symptoms shown are associated with alfalfa mosaic virus (AMV) (**A**–**C**), beet western yellows virus (BWYV) (**D**–**F**), pepper mild mottle virus (PMMoV) (**G**–**I**), potato yellow dwarf/constricta yellow dwarf virus (PYDV/CYDV) (**J**–**L**), beet curly top virus (BCTV) (**M**–**O**), tomato yellow leaf curl virus (TYLCV) (**P**–**R**), and pepper yellow dwarf virus (BCTV-PeYDV) (**S**–**U**).

Plants infected with alfalfa mosaic virus (AMV) exhibited yellow leaf blotching and mottling (Figure 2A–C). Infection by beet western yellows virus (BWYV) resulted in yellowing, leaf deformation, and mottling (Figure 2D–F). Symptoms of pepper mild mottle virus (PMMoV) included leaf cupping, deformation, and mosaic patterns (Figure 2G–I). In plants infected with potato yellow dwarf virus/constricta yellow dwarf virus (PYDV/CYDV), severe yellowing, stunting, and interveinal chlorosis were observed (Figure 2J–L). Beet curly top virus (BCTV) caused rubbery leaves, yellowing, and stunted growth (Figure 2M–O). Infection with tomato yellow leaf curl virus (TYLCV) led to leaf cupping, curling, and yellowing (Figure 2P–R). Finally, plants infected with pepper yellow dwarf virus (BCTV-PeYDV) showed symptoms of leaf deformation and yellowing (Figure 2S–U).

Symptomatic plants showed severe effects of virus infection. Pepper plants infected with DNA viruses exhibited a marked reduction in both the number and size of fruits compared to pepper plants infected with RNA viruses. In some cases, infection with DNA viruses, such as BCTV, resulted in no fruit production or the formation of small, unmarketable fruits, leading to nearly complete yield loss. Additionally, mixed infections involving RNA viruses were associated with more severe leaf deformation (Figure 2G).

### 3.2. HTS Total Read Count and Contigs Assembled

Among the 63 samples processed by HTS, the total read count ranged from 15.5 and 285 million (Supplementary Table S2). The average read count was 33.5 million, with a median of 22.5 million reads. The number of assembled contigs ranged from 17,407 to 202,526 with a mean of 55,282 and a median of 47,145 (Supplementary Table S2).

### 3.3. Viruses Identified by HTS from Pepper

A total of 13 unique viruses and one viroid were identified in pepper samples through HTS (Table 2). The detected viruses included single-stranded RNA (+ssRNA and -ssRNA) (n = 8 or 61%), double-stranded RNA (dsRNA) (n = 2 or 15%), single stranded DNA (ssDNA) (n = 2 or 15%), and double-stranded DNA (dsDNA) (n = 1 or 7%). The Insect narna-like virus 1 contigs were the most frequently detected, with 48 occurrences across all samplings. Pepper cryptic virus 1 and 2 (PCV-1 and PCV-2) were each detected 41 times though not always together. Additionally, a potentially novel rhabdovirus and a viroid were each identified from pepper samples collected in Cherokee and Caddo counties, respectively.

Nucleotide sequence variation was briefly compared among isolates of the same virus detected during our surveys, based on the acquired contig sequences. PCV-1 and PCV-2 showed minimal sequence variation from one another, with identities ranging from 97 to 100%. Similarly, PMMoV isolates showed 99–100% identity, while the BCTV-PeYDV strain ranged from 98 to 100%. PYDV/CYDV exhibited identities between 98 and 100%. In contrast, BCTV and BPEV showed greater sequence variation with identities from 78 to 100% and 86–100%, respectively.

### 3.4. Viruses Identified by HTS from Non-Pepper Hosts

Contigs of four viruses were identified in non-pepper crops growing near pepper fields but were not detected directly from pepper plants (Table 3). All viruses detected in non-pepper hosts possessed single-stranded RNA genomes and were detected in Cherokee County. Each of these viruses was detected only once. Blackberry chlorotic ringspot virus (BCRV) and ocimum basilicum RNA virus 2 (ObRV2) were identified in basil and squash plants grown adjacent to pepper. Pokeweed mosaic virus (PkMV) was detected from American pokeweed (*Phytolacca Americana*), located less than 100 feet from pepper plantings. Potato leafroll virus (PLRV) was identified in tomato (*Solanum lycopersicum*). Additionally, a viroid-like sequence, identified as Citrus exocortis Yucatan viroid, was detected in tomato (*Solanum lycopersicum*) from Tulsa County.

In total, 17 viruses or virus-like sequences and two viroid-like sequences (19 in all) were detected by HTS from both pepper and non-pepper hosts. The majority of viruses have single-stranded RNA genomes (12 out of 17, or 70%), while the remaining consisted of double-stranded RNA (2/17 or 11.7%), single-stranded DNA (2/17 or 11.7%), and double-stranded DNA genomes (1/17 or 5.8%). Throughout the survey period, HTS detected 230 virus isolates during 2021–2022 growing seasons (Table 4).

### 3.5. Occurrence of Viruses in 2021 and 2022

**Figure 3:**
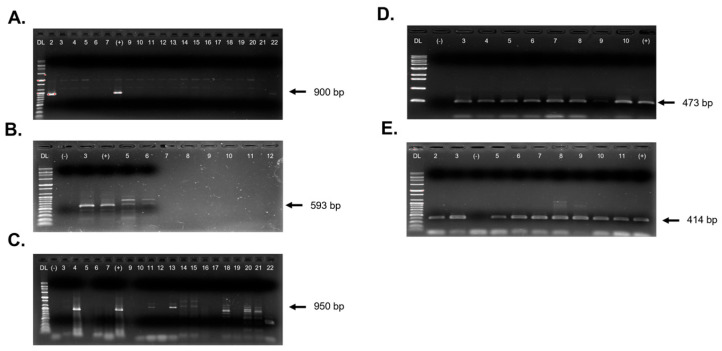
Detection of RNA viruses using RT-PCR assays, visualized on 1% agarose gel electrophoresis. (**A**) alfalfa mosaic virus (AMV) amplified with primers AMV-900F and AMV-900R (expected amplicon size: 900 bp); (**B**) beet western yellows virus (BWYV) with primers BWYV-593F and BWYV-593R (expected size: 593 bp); (**C**) cucumber mosaic virus (CMV) with primers CMV-F and CMV-R (expected size: 950 bp); (**D**) pepper mild mottle virus (PMMoV) with primers PMMoV-CPF and PMMoV-CPR (expected size: 473 bp); (**E**) potato yellow dwarf virus/constricta yellow dwarf virus (PYDV/CYDV) with primers PYDV-414F and PYDV-414R (expected size: 414 bp). DL indicates the DNA ladder where (**A**–**C**,**E**) used the 100 bp Plus DNA ladder, and **D**) used the 1 kb DNA ladder. (+) denotes the positive control for each virus; (−) denotes the negative control, which included either RNA from healthy tissue or water.

**Figure 4:**
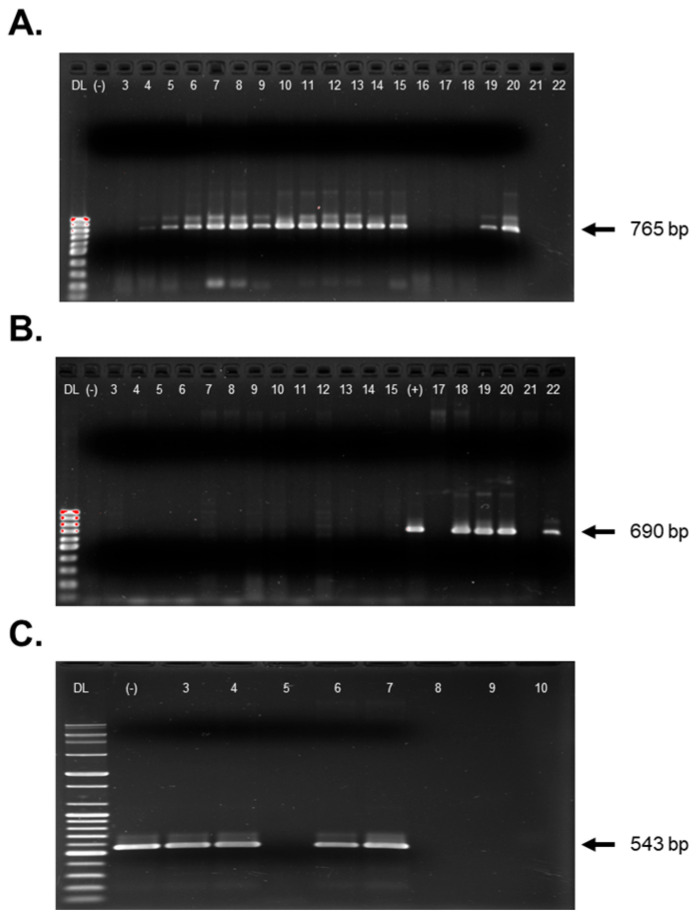
Detection of DNA viruses identified by HTS, confirmed by PCR and visualized via agarose gel electrophoresis. (**A**) beet curly top virus (BCTV) amplified with BCTV-CPF and BCTV-CPR primers (expected amplicon size: 765 bp); (**B**) pepper yellow dwarf virus (PeYDV, a strain of BCTV) with PeYDV-690F and PeYDV-690R primers (expected size: 690 bp); (**C**) Tomato yellow leaf curl virus (TYLCV) with TYLCV-543F and TYLCV-543R primers (expected size: 543 bp). DL indicates the DNA ladder: (**A**,**B**) used the 1 kb DNA ladder, while (**C**) used the 100 bp Plus DNA ladder. (+) represents the positive control for the respective virus, while (−) represents the negative control, containing either DNA from healthy tissue or water.

All samples (n = 310) were tested using RT-PCR and PCR assays targeting seven plant viruses: AMV, BCTV, CMV, PMMoV, PYDV/CYDV, and TYLCV. These viruses were selected based on their known association with economic losses in pepper and their higher detection frequency through HTS. Each assay included virus specific positive and negative controls to ensure accuracy. Focus was placed on five RNA viruses (Figure 3), two DNA viruses, and one specific virus strain (Figure 4). Agarose gels electrophoresis was performed using either the 100 bp Plus DNA ladder (Bioneer, Yuseong-gu, Daejeon, South Korea) or the 1 kb DNA ladder (Bioneer, South Korea) as molecular size markers.

In the 2021 growing season, PCR assays confirmed the presence of AMV, BCTV, BCTV-PeYDV, BWYV, CMV, PMMoV, and PYDV/CYDV in samples collected from Caddo County (Table 5). No virus-like symptoms were observed on pepper plants in Atoka, Cherokee, or Muskogee counties during this time. TYLCV was detected only in Tulsa County, from both pepper and tomato samples. Greer County was not surveyed in the 2021 growing season. Overall virus occurrence revealed that insect narna-like virus and pepper cryptic viruses (PCV-1 and PCV-2) were present in more than 50% of all HTS samples. Among the economically important viruses, PMMoV was the most prevalent, with 19 detections, all originating from Caddo County, making it the dominant virus identified (Table 5). BCTV (n = 10) and AMV (n = 8) were also detected at relatively high frequencies. The remaining viruses (CMV, BWYV, and PYDV/CYDV) were detected at lower frequencies (2–8 detections each), all of which also originated from Caddo County.

**Figure 5:**
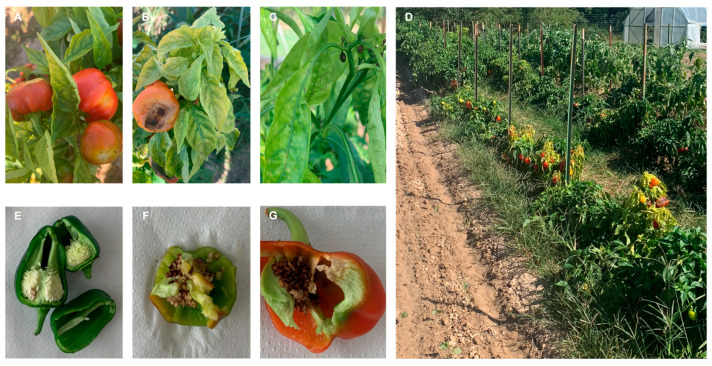
Symptoms on pepper caused by PYDV/CYDV. (**A**) fruit deformation on bell pepper, (**B**) apical yellowing and fruit necrosis, (**C**) flower bud necrosis, (**D**) symptoms associated with plants in the field, (**E**) presumably healthy pepper fruit, and (**F**,**G**) fruit from PYDV/CYDV infected plants with deformed fruit and necrotic seeds.

In 2022, BCTV was found to be the most frequently detected virus (n = 43) with detections primarily from Caddo and Greer counties (Table 6). The BCTV-PeYDV strain was identified 22 times, often in conjunction with BCTV infection. Interestingly, AMV, BWYV, CMV, PMMoV, and PYDV/CYDV were not detected in Caddo County in 2022. CMV and PYDV/CYDV were detected in Cherokee County, with one and twenty-nine detections, respectively. The effects of PYDV/CYDV infection on fruit, seed, flower, and plant in Cherokee County were severe (Figure 5). TYLCV was detected exclusively in Tulsa County as in the 2021 surveys. In contrast to 2021, insect narna-like virus 1, PCV-1, and PCV-2 accounted for a smaller proportion (~25%) of total positive samples. Overall, the most frequently detected viruses across 2021 and 2022 were TYLCV (n = 30), PYDV/CYDV (n = 31), and BCTV (n = 53). Including the BCTV-PeYDV strain, the total number of BCTV-related isolates reached 77.

### 3.6. Virus Detection Rates for 2021 and 2022

**Figure 6:**
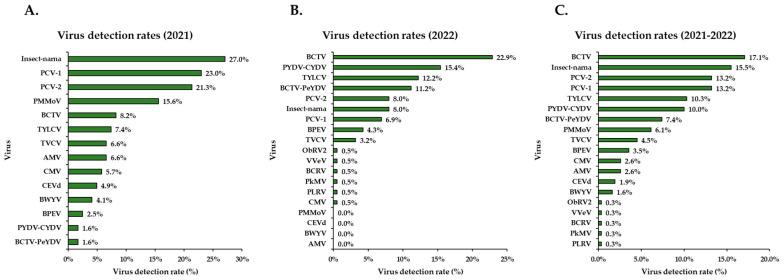
Virus detection rates across all surveys based on HTS and RT-PCR assays. (**A**) Detection rate of viruses identified in 2021. (**B**) Detection rate of viruses identified in 2022. (**C**) Overall detection rate combining data from 2021 and 2022. **Abbreviations:** AMV = alfalfa mosaic virus; BCTV = beet curly top virus; BCTV-PeYDV = pepper yellow dwarf virus (a strain of BCTV); BWYV = beet western yellows virus; BPEV = bell pepper alphaendornavirus; BCRV = blackberry chlorotic ringspot virus; CMV = cucumber mosaic virus; Insect-narna = Insect narna-like virus 1; PMMoV = pepper mild mottle virus; PkMV = pokeweed mosaic virus; PLRV = potato leafroll virus; PYDV/CYDV = potato yellow dwarf/constricta yellow dwarf virus; PCV-1 = pepper cryptic virus 1; PCV-2 = pepper cryptic virus 2; TYLCV = tomato yellow leaf curl virus; TVCV = tobacco vein clearing virus; VVeV = viola verecunda virus 1; CEVd = citrus exocortis viroid; ObRV2 = ocimum basilicum RNA virus 2.

In 2021, detection rates showed that PMMoV and BCTV were present in 16% and 8% of the total samples collected (n = 122), respectively (Figure 6A). Cryptic viruses (PCV-1, PCV-2, and BPEV) were collectively detected in approximately ~47% of the samples that year.

In 2022, BCTV was detected more frequently, with a 23% detection rate (Figure 6B). TYLCV and the BCTV-PeYDV strain were identified in 12% and 11% of samples, respectively, while PYDV/CYDV was found in 15% of all samples collected (n = 188). In contrast to 2021, cryptic viruses were less prevalent in 2022, occurring in only 19% of samples.

Across both years (2021–2022), BCTV was detected in 17% of the total samples collected (n = 310), indicating its presence in nearly one out of every five samples (Figure 6C). When including BCTV-PeYDV strain, which accounted for 7% of detections, the overall proportion of BCTV-related detections rose to 24% or nearly one in four samples. TYLCV and PYDV/CYDV each had an overall detection rate of 10%, while cryptic viruses (PCV-1, PCV-2, and BPEV) were found in 30% of all samples collected during the study.

Detection rates among individual surveys were also examined. Notably BCTV was detected in 91% of samples in one survey, PYDV/CYDV in 84%, TYLCV in 57%, and PMMoV in 47% of samples.

### 3.7. Mixed Viral Infections

Based on HTS data, mixed infections involving multiple viruses as well as virus-viroid combinations were frequently observed. In 9 out of 63 (14%) samples, five different viruses were detected within a single sample. Additionally, mixed infections of four viruses were found in 15 out of 63 samples (23.8%). Furthermore, mixed infections involving economically important viruses or virus strains were identified in 17 cases (17 out of 63 samples, or 27%) (Supplementary Table S3). The most frequently associated RNA virus in these mixed infections was CMV, detected in 8 instances. Mixed-infections involving BCTV and the BCTV-PeYDV strain occurred 7 times in the HTS dataset. PCR screening revealed an additional 10 cases of mixed infections involving both BCTV and its PeYDV strain.

Overall, 8.7% of all samples collected (n = 310) contained more than one economically important virus or virus strain. Among the mixed infection cases, 3 out of 27 (11%) involved three different viruses. However, only 0.96% of the total samples set exhibited mixed infections with three economically important viruses.

## 4. Discussion

The detection of viral agents in this study was made possible by advances in sequencing technologies, particularly high-throughput sequencing (HTS). By applying HTS to analyze the pepper virome in Oklahoma, we were able to identify known, previously unreported, and potentially novel viruses and viroids. This approach also enabled the detection of mixed infections, which were often associated with more severe disease symptoms during field surveys.

Several economically important viruses identified in this study (AMV, BCTV, BWYV, CMV, PMMoV, PYDV/CYDV, and TYLCV) have historically caused significant disease and economic losses in pepper and other crops [31,32,33]. Among these, BCTV emerged as the most prevalent DNA virus in pepper, with 77 positive cases confirmed through both PCR and HTS. BCTV is known to infect more than 300 plant species [34], posing a threat not only to pepper but also to other economically valuable crops, particularly in western Oklahoma where the virus was frequently detected.

Our lab detected BCTV in 2018 from Caddo and Blaine counties, marking it as the first DNA virus from pepper in Oklahoma [35]. In the current study, BCTV and its PeYDV strain were detected in two western counties (Caddo and Greer). The observed genetic variability within BTCV populations suggests the potential for recombination events [36] which could influence virus evolution and virulence; however, this remains an area for future investigation. These findings indicate a wider geographical distribution of BTCV across Western Oklahoma than previously recognized. This underscores the need for expanded surveillance in additional counties to better understand the distribution and diversity of BCTV and its associated strains.

TYLCV was the second most frequently detected DNA virus with 32 occurrences identified in pepper and tomato samples. TYLCV was first reported in Oklahoma by our lab and based on our surveys, remains localized to Tulsa County [37]. Ongoing surveillance is essential to determine whether the virus will remain confined to this region, expand into other counties, or is already present but not detected elsewhere. During our surveys in Tulsa County, whiteflies were frequently observed on pepper and tomato plants potentially facilitating virus spread. Although whiteflies were also observed in other counties of Oklahoma, TYLCV was not identified outside Tulsa County.

Among RNA viruses of economic significance, the most commonly detected was PYDV/CYDV with 31 occurrences, followed by PMMoV (n = 19). In the 2021 growing season, we reported the presence of PYDV for the first time in Caddo County Oklahoma [38]. Initially, these detections were attributed to PYDV; however, recently updated nomenclature on NCBI suggests that the Oklahoma isolates are more accurately classified as constricta yellow dwarf virus (CYDV), a close relative of PYDV. Ongoing analyses aim to resolve this taxonomic discrepancy. Accordingly, the acronym PYDV/CYDV is used throughout this text to reflect the current understanding.

Unfortunately, PYDV/CYDV has now been identified in both Caddo and Cherokee counties which are approximately 230 miles apart. In 2022, further investigation confirmed the presence of PYDV/CYDV in Cherokee County. Expanding the geographic scope of surveys will be necessary to determine the full extent of PYDV/CYDV distribution in Oklahoma and potentially in neighboring states.

PMMoV has been extensively studied since its emergence as a viral pathogen affecting pepper crops. In addition to its agricultural relevance, PMMoV continues to be investigated for its role in water quality assessment [39] and as an indicator of fecal pollution [40]. The virus has been detected in nontraditional irrigation sources, where elevated concentrations of viral pathogens, including PMMoV, have been observed [41].

The present study focused on virus detection in crop samples rather than irrigation sources; therefore, water testing for PMMoV was not conducted. Nonetheless, incorporating water source analyses into future research could yield valuable insights, particularly regarding the presence of PMMoV in western Oklahoma. Interestingly, PMMoV was detected only during the growing seasons of 2021 and remains localized to Caddo County in western Oklahoma based on current survey data.

Currently, definitive evidence regarding the initial introduction of these viruses into Oklahoma is lacking. However, future investigations may help formulate hypotheses regarding their pathways of entry. One plausible explanation is the introduction of virus-infected germplasm; alternatively, the expanded geographic range of insect vectors may also play a role [42]. It is important to note that virus-associated vectors were consistently documented throughout our surveys. A total of 30 individual surveys were conducted, during which aphids, leafhoppers, whiteflies, and mealybugs were observed in approximately ~40% of cases. In some instances, multiple vector species were co-feeding on the same plant, potentially increasing the risk of viral transmission and mixed infection.

Of the 63 libraries prepared for HTS, pepper cryptic viruses (PCV-1 and PCV-2) were detected from most samples analyzed (41 out of 63, or approximately 65%). This indicates that while pepper cryptic viruses were not found infecting all peppers plants within our study, they were found infecting most of those surveyed. Samples infected only with cryptic viruses appeared healthy as did plants infected solely with BPEV, which showed no visible symptoms. As a result, PCR assays were not developed for the cryptic viruses (PCV-1, PCV-2, and BPEV) in this study. Recently, our laboratory published a study on BPEV detection and phylogenetics as an extension of our work on the pepper virome [43]. We aim to continue this line of research with similar studies on PCV-1 and PCV-2.

Therefore, efforts were focused on viruses of greater economic significance that are known to cause noticeable disease symptoms in pepper. Because virus occurrence and detection rates were based on both HTS and PCR data, the cryptic viruses were underrepresented. If PCR assays were available for their detection, these viruses would have accounted for a larger share of the total viruses identified. Pepper cryptic viruses are vertically transmitted from parent to offspring via seed [44], a trait also observed in BPEV [16]. A beneficial quality of PCV-1 was reported in a previous study to reduce herbivory by aphids [45]. Likewise, BPEV does not currently induce visible pathogenic effects in infected plants [16]. These characteristics support the inclusion of these viruses in the present study, as their interactions with pepper hosts remain poorly understood. Future studies on these viruses are necessary to explore their potential role in limiting insect herbivory and to uncover additional effects, whether beneficial or detrimental, they may have on the host plant.

Viroids are known to be transmitted through seed and propagative material [46]. Citrus exocortis viroid (CEVd) which has been reported to induce stress in tomato plants [47] has also been detected in pepper seed lots [48]. In this study, CEVd-related sequences were detected which is notable given the limited investigation of CEVd in natural pepper infection. BLAST analysis identified one of the sequences as citrus exocortis Yucatan viroid (MZ463745.1). According to the species demarcation criterion for the *Pospiviroid* genus, a novel species must share less than 90% sequence identity with existing species and exhibit distinct biological properties [49]. The citrus exocortis Yucatan viroid sequence detected in this study showed 98% identity with previously reported CEVd sequences, supporting its classification as a CEVd isolate. Interestingly, another partial viroid sequence identified in pepper showed only 86.5% identity to a different CEVd isolate (OR024670.1), indicating not only the potential for CEVd to infect pepper, but also the presence of a novel CEVd-related viroid in Oklahoma.

HTS analysis of two distinct samples revealed that cryptic viruses and CEVd were the only virus-related agents present. The affected plants exhibited severe chlorosis and leaf curling. The observed symptoms in plants harboring CEVd-like sequences closely resembled those previously reported in tomato plants infected with CEVd [50]. This correlation between HTS sequence data and symptomatology suggests that CEVd, along with a potentially novel *Pospiviroid* may be contributing to disease development in some pepper plants in this study. Comprehensive molecular and biological characterization of CEVd would be invaluable in supporting its etiological role in the observed disease symptoms. In addition, further characterization of the putative novel *Pospiviroid* could provide important insights into its identity, biology, and potential impact on pepper health.

The apparent detection of a potentially novel RNA-dependent RNA polymerase (RdRP) gene sequence associated with a rhabdovirus is of significant scientific interest. In a recent virome study, contigs corresponding to novel rhabdoviruses were identified [13]. Our analyses yielded a similar result, with a 4767 nucleotide contig exhibiting notable sequence identity to Viola verecunda virus 1 (Genbank accession: BK014333.1). One contig (4767 nt) was evaluated by BLASTn analysis revealing a 73% nucleotide identity (BK014333.1), while BLASTx showed a 68% amino acid identity with the same Viola verecunda virus 1 isolate. Viola verecunda virus 1 remains an unclassified member of the family *Rhabdoviridae*, but a common demarcation criterion within the subfamily *Betarhabdovirinae* (plant virus-related taxa), is a complete nucleotide sequence or L gene of less than 75% to classify a virus as a novel species [51]. However, because a complete genome sequence was not obtained, it remains uncertain whether the detected contig represents a divergent isolate of an existing rhabdovirus. In the field setting, the Viola verecunda-like virus identified in our study exhibited virus-like symptoms in contrast to presumably healthy plants. This preliminary discovery requires further molecular and biological characterization to determine the taxonomic status and pathogenic potential of the virus.

Other viruses associated with pepper that were identified by HTS, included insect narna-like virus-1 and tobacco vein clearing virus (TVCV). The biological role of insect narna-like virus-1 remains unclear, a gap further compounded by the limited research currently available on this virus. Several sequences are available in NCBI which have been identified from *Frankliniella occidentalis* (e.g., MN764145 and MW297845) as the reported host. The sequences which aligned to these accessions shared greater than 95% identity in most cases. Given both the tentative classification of these virus-like sequences and their recurring detection, further investigation is warranted.

TVCV was detected less frequently via HTS. In most instances, the assembled contigs were short and exhibited low average sequence coverage. However, some contigs were of greater length and showed higher coverage, which precluded the dismissal of TVCV as an assembly artifact or chimeric sequence. Additionally, one field sample showed distinct vein clearing symptoms and HTS analysis revealed the presence of TVCV along with cryptic viruses (PCV-1 and PCV-2) as the only detected virus-like sequences. Further confirmation of TVCV’s presence is necessary to substantiate its involvement in symptom expression. Nevertheless, compared to historically significant and economically impactful viruses detected in this study, TVCV and insect narna-like virus 1 were considered of lower priority for immediate follow-up.

While the primary focus of this study was on viruses affecting pepper, HTS also revealed the presence of viruses in other crop species. More recently, CMV was detected in spinach (April 2023) from a field where CMV had been identified in pepper the previous year (Supplementary Figure S1). CMV has been previously demonstrated to be seed transmissible in pepper [52] and seed transmission is likely the source of infection in this case, supported by the observations of symptomatic volunteer pepper seedlings growing near the spinach crop. According to the outbreak, it resulted in an estimated economic loss of $500–1000. Additional viruses detected in non-pepper crops included PLRV from tomato, PkMV from pokeweed, and both BCRV and ObRV2 from basil and squash. These detections occurred in crops grown adjacent to pepper fields and may lead to subsequent disease outbreaks and economic losses in pepper and other susceptible crops. These findings underscore the critical importance of monitoring viral pathogens not only in the target host crop but also in surrounding vegetation. The proximity of alternative hosts may serve as reservoirs for virus maintenance and spread, thereby increasing the risk to economically important crops under favorable conditions.

For broader context, the 2017 United States Census of Agriculture reported that Oklahoma ranked fourth nationally in the total number of farms [53]. These farms support a diverse range of agricultural commodities, including numerous economically significant crops. The emergence and spread of plant viruses in Oklahoma is therefore of increasing concern, with potential implications for the sustainability and productivity of the state’s agricultural systems. Although the primary focus of this was on virus detection in pepper, several additional viruses were identified, that may pose a threat to other important crops. For example, the detection of CMV in spinach highlights the broader agricultural risk associated with viral pathogens. Factors that contribute to the emergence and dissemination of plant viruses include monoculture cultivation, the movement of infected germplasm, and the ongoing need for expanding agriculture [54]. The findings of this study extend beyond the interests of pepper growers. They provide valuable insights for producers cultivating a wide range of crops across Oklahoma. Importantly, the identification of these viruses lays the groundwork for developing proactive disease management strategies aimed at mitigating future outbreaks, thereby supporting the long-term resilience of Oklahoma’s agricultural sector.

## 5. Conclusions

Virome studies aimed at understanding the diversity and presence of plant viruses remain limited in Oklahoma. In this study, a total of 17 viruses and two viroid were detected from pepper plants, as well as other crops and weeds. Some of the results presented are of a putative nature and require subsequent characterization before authentic conclusions can be drawn. While certain results are preliminary, their implications are highly important to local growers. Several of the economically important viruses identified have historically caused severe disease outbreaks and substantial crop losses in pepper and other hosts worldwide. In Oklahoma, however, their impact may have gone largely unrecognized due to the absence of focused research.

The detection of these pathogens in pepper fields provides a foundation for the development of more effective and targeted management strategies. For instance, growers in Tulsa County may implement more rigorous methods to prevent whitefly feeding and colonization. Overall, this virome-based approach is indispensable for minimizing potential crop losses in pepper and offers valuable insights for Oklahoma pepper growers. The findings from this study will support increased vigilance and preparedness against sporadic or epidemic viral infection in local pepper fields.

## Figures and Tables

**Table 1:** Number of samples collected from pepper plants and adjacent non-pepper hosts during the 2021 and 2022 growing seasons in Oklahoma.

County	Basil	Blackberry	Insect	Pepper	Potato	Squash	Tomato	Miscellaneous ^1^	Total
Atoka	0	0	0	3	0	4	0	0	7
Caddo	0	0	0	124	0	0	0	0	124
Cherokee	2	3	1	49	5	7	2	2	71
Greer	0	0	5	32	0	0	0	2	39
Muskogee	0	0	0	2	0	0	0	0	2
Tulsa	0	0	0	18	0	3	31	15	67
Total	2	3	6	226	5	14	33	21	310

^1^ This includes many different species of plants near pepper fields.

**Table 2:** Representative viruses and virus-like agents detected by HTS from pepper (*Capsicum annuum*).

No.	Virus/Viroid Name	Family	Genome	Total Read Count	Average Coverage	Contig Length	Percent nt Identity (%)	Reference Accession ^1^
1	Alfalfa mosaic virus	Bromoviridae	ssRNA (+)	1,111,851	22,699	3601	99.6	MH332897.1
2	Beet western yellows virus	Solemoviridae	ssRNA (+)	6030	328	1349	97.5	LC428355.1
3	Bell pepper endornavirus	Endornaviridae	ssRNA (+)	42,322	256	12,140	99.8	MN073197.1
4	Cucumber mosaic virus	Bromoviridae	ssRNA (+)	475,944	12,330	2843	98.8	AF416900.1
5	Pepper mild mottle virus	Virgaviridae	ssRNA (+)	756,359	8825	6350	99.6	AB000709.2
6	Potato yellow dwarf virus/Constrica yellow dwarf virus	Rhabdoviridae	ssRNA (-)	3,353,898	19,711	12,624	90.7	NC_076163.1
7	Viola verecunda virus 1	Rhabdoviridae	ssRNA (-)	17,107	265	4767	73.3	BK014333.1
8	Pepper cryptic virus 1	Partitiviridae	dsRNA	27,045	1292	1547	100	KY923702.1
9	Pepper cryptic virus 2	Partitiviridae	dsRNA	177,086	8311	1531	99.8	KY923704.1
10	Beet curly top virus	Geminiviridae	ssDNA	62,068	1541	2926	98.8	AY548948.1
10.1	BCTV-pepper yellow dwarf virus	Geminiviridae	ssDNA	145,273	3590	2990	99.0	EU921828.1
11	Tomato yellow leaf curl virus	Geminiviridae	ssDNA	23,392	617	2781	99.8	ON321843.1
12	Tobacco vein clearing virus	Caulimoviridae	dsDNA	11,915	1438	579	84.5	NC_003378.1
13	Citrus exocortis viroid	Pospiviroidae	ssRNA	6033	1863	215	86.5	OR024670.1
14	Insect narna-like virus 1	Narnaviridae	ssRNA	128,534	3111	3057	96.7	MN764145.1

^1^ Refers to the closest matching accession number by BLASTn analysis.

**Table 3:** Representative viruses identified by HTS in other crops adjacent to pepper fields.

No.	Virus	Family	Genome	Host	Total Read Count	Average Coverage	Contig Length	Percent nt Identity (%)	Reference Accession
1	Blackberry chlorotic ringspot virus	Bromoviridae	ssRNA (+)	Basil, Squash	74,148	2404	2278	98.7	JX429895.1
2	Pokeweed mosaic virus	Potyviridae	ssRNA (+)	Pokeweed	5,593,771	42,963	9629	97.7	MG189944.1
3	Potato leafroll virus	Solemoviridae	ssRNA (+)	Tomato	4994	164	2236	97.5	AY138970.1
4	Ocimum basilicum RNA virus 2	Mitoviridae	ssRNA	Basil, Squash	200,707	5349	2770	97.6	NC_035463.1

**Table 4:** Number of isolates identified per virus or virus-like agent by HTS analysis.

No.	Virus/Viroid Name/s	Abbreviation	Number of Isolates Detected by HTS
1	Insect narna-like virus 1	-	48
2	Pepper cryptic virus 1	PCV-1	41
3	Pepper cryptic virus 2	PCV-2	41
4	Beet curly top virus	BCTV	15
5	Tobacco vein clearing virus	TVCV	14
6	Bell pepper alphaendornavirus	BPEV	11
7	Potato yellow dwarf virus/constricta yellow dwarf virus	PYDV/CYDV	11
8	Pepper yellow dwarf virus	BCTV-PeYDV	9
9	Cucumber mosaic virus	CMV	8
10	Alfalfa mosaic virus	AMV	7
11	Citrus exocortis viroid	CEVd	6
12	Pepper mild mottle virus	PMMoV	6
13	Beet western yellows virus	BWYV	5
13.1	Tomato yellow leaf curl virus	TYLCV	3
14	Blackberry chlorotic ringspot virus	BCRV	1
15	Ocimum basilicum RNA virus 2	ObRV2	1
16	Pokeweed mosaic virus	PkMV	1
17	Potato leafroll virus	PLRV	1
18	Viola verecunda virus 1	VVeV1	1
		Total	230

**Table 5:** Viruses detected through HTS and PCR assays (virus occurrence) across counties surveyed during the 2021 growing season.

County	No. Samples	AMV	BCTV	BCTV-PeYDV	BWYV	CMV	PMMoV	PYDV-CYDV	TYLCV	Total Positive
Atoka	7	0	0	0	0	0	0	0	0	0
Caddo	83	8	10	2	5	7	19	2	0	53
Cherokee	6	0	0	0	0	0	0	0	0	0
Greer	NS	NS	NS	NS	NS	NS	NS	NS	NS	0
Muskogee	2	0	0	0	0	0	0	0	0	0
Tulsa	24	0	0	0	0	0	0	0	7	9
2021 Total	122	8	10	2	5	7	19	2	7	60

Note: All viruses were detected in pepper samples, except those from Tulsa County, which included three pepper and four tomato samples. Abbreviations: NS = Not surveyed; AMV = alfalfa mosaic virus; BCTV = beet curly top virus; BCTV-PeYDV = pepper yellow dwarf virus (a strain of BCTV); BWYV = beet western yellows virus; CMV = cucumber mosaic virus; PMMoV = pepper mild mottle virus; PYDV/CYDV = potato yellow dwarf/constricta yellow dwarf virus; TYLCV = tomato yellow leaf curl virus.

**Table 6:** Viruses detected through HTS and PCR assays (virus occurrence) across counties surveyed during the 2022 growing season.

County	No. Samples	AMV	BCTV	BCTV-PeYDV	BWYV	CMV	PMMoV	PYDV-CYDV	TYLCV	Total Positive
Atoka	NS	NS	NS	NS	NS	NS	NS	NS	NS	0
Caddo	41	0	15	11	0	0	0	0	0	26
Cherokee	65	0	0	0	0	1	0	29	0	30
Greer	39	0	28	11	0	0	0	0	0	39
Muskogee	NS	NS	NS	NS	NS	NS	NS	NS	NS	0
Tulsa	43	0	0	0	0	0	0	0	23	23
2022 Total	188	0	43	22	0	1	0	29	23	118
2021 and 2022	310	8	53	24	5	8	19	31	30	178

**Note:** All viruses detected by PCR were from pepper samples, except those collected in Tulsa County. In Tulsa County, all positive detections were from tomato leaf tissue and were PCR-confirmed for the presence of tomato yellow leaf curl virus (TYLCV). **Abbreviations:** NS = Not surveyed; AMV = alfalfa mosaic virus; BCTV = beet curly top virus; BCTV-PeYDV = pepper yellow dwarf virus (a strain of BCTV); BWYV = beet western yellows virus; CMV = cucumber mosaic virus; PMMoV = pepper mild mottle virus; PYDV/CYDV = potato yellow dwarf/constricta yellow dwarf virus; TYLCV = tomato yellow leaf curl virus.

## Data Availability

Data discussed throughout the text can be made available at the reader’s request.

## Supplementary Materials

The following supporting information can be downloaded at: https://www.mdpi.com/article/10.3390/pathogens14101035/s1, Figure S1: CMV infection in spinach. (A) mosaic and early infection by CMV. (B) Stunting and leathery leaf during more severe and later infection. (C) Healthy (top) and infected (bottom) spinach. Samples collected were subsequently confirmed by PCR using specific primers; Table S1: Primers used in RT-PCR and PCR assays were developed to screen samples for the predominant RNA and DNA viruses (References [28,32,38,49,55] are cited in Table S1); Table S2: Sequence reads and assembles contigs for each sample processed by Illumina HTS; Table S3: Mixed viral infections of viruses and virus strains detected by HTS.
