# Supplementary material for: Characterization of the Pepper Virome in Oklahoma Reveals Emerging RNA and DNA Viruses
Source: Pathogens. 2025 Oct 13;14(10):1035. doi: 10.3390/pathogens14101035 (PMC12567154; doi:10.3390/pathogens14101035)
Supplement: Supplementary file 1 [file pathogens-14-01035-s001.zip › pathogens-3840291-supplementary.pdf]

Supplementary Materials

Supplementary Table S1. Primers designed and used in RT-PCR and PCR assays for screening the predominant RNA and DNA viruses in the samples.

Primer Name	Virus	Sequence (5-3)	Gene	Size (bp)	Reference
**AMV-900F**	AMV[1]	GTCCGCGATCTCT TAAAT	CP	900	[28]
**AMV-900R**	AMV[1]	TCTCTCGACCCAA ACTTC	CP		
**BCTV-CPF**	BCTV[2]	ATGAGGAAATAT ACAAGAAA	CP	765	[32]
**BCTV-CPR**	BCTV[2]	TTAATAAAAATA GCATCTACA	CP		
**BWYV-593F**	BWYV[1]	GAAGAACAATCA ATGGG	CP, MP, AT	593	Designed in this study
**BWYV-593R**	BWYV[1]	TACCTATTTGGGG TTGT	CP, MP, AT		
**CMV-F**	CMV[1]	GGCTGCAGTGGTC TCCTT	CP	950	[49,55]
**CMV-R**	CMV[1]	GAGTCGAGTCAT GGACAAATC	CP		
**PepYDV-690F**	PepYDV[2]	CTCCCAATTATAT GACCCAA	CP, REP, TRaP	690	Designed in this study
**PepYDV-690R**	PepYDV[2]	CAAGTGCTGGAT GGATTT	CP, REP, TrAP		
**PMMoV-CPF**	PMMoV[1]	ATGGCTTACACAG TTTCCA	CP	473	[32]
**PMMoV-CPR**	PMMoV[1]	TTAAGGAGTTGTA GCCCA	CP		
**PYDV-414F**	PYDV[1]	GCAGACAAGAAC AAAGAG	RdRP	414	[38]
**PYDV-414R**	PYDV[1]	GGGTTGCATTCTG ACTG	RdRP		
**TYLCV-543F**	TYLCV[2]	ACGCATGCCTCTA ATCCAGTGTA	CP	543	Designed in this study
**TYLCV-543R**	TYLCV[2]	CCAATAAGGCGT AAGCGTGTAGAC	CP		

[1] RNA virus, [2] DNA virus

AMV = alfalfa mosaic virus, BCTV = beet curly top virus, BWYV = beet western yellows virus, CMV = cucumber mosaic virus, PepYDV = pepper yellow dwarf virus (strain of BCTV), PMMoV = pepper mild mottle virus, PYDV = potato yellow dwarf/constricta yellow dwarf virus, TYLCV = tomato yellow leaf curl virus, CP = coat protein, MP = movement protein, REP = replication enhancer protein, TrAP = transcriptional activator protein, RdRP = RNA-dependent RNA polymerase

Supplementary Table S2. Sequence reads and assembled contigs generated for each sample using high-throughput sequencing (HTS).

Library ID	Host	County	Total read count	Average length of reads	Contigs assembled	Average length of contigs
**1**	Pepper[2]	Caddo	20,805,488	73.2	38,725	334
**2**	Pepper	Caddo	77,731,117	73.1	95,557	353
**3**	Pepper	Caddo	17,408,110	72.8	29,271	326
**4**	Pepper	Caddo	42,791,838	73.4	58,668	333
**5**	Pepper	Caddo	21,841,730	73.5	22,785	315
**6**	Pepper	Caddo	285,277,288	73.4	202,526	372
**7**	Pepper	Tulsa	63,488,734	73.6	93,234	330
**8**	Tomato[3]	Tulsa	19,037,059	73.6	35,419	351
**9**	Pepper	Caddo	22,045,381	73.5	39,592	337
**10**	Pepper	Caddo	17,756,794	73.5	28,891	333
**11**	Pepper	Caddo	45,780,855	73.5	63,928	348
**12**	Pepper	Caddo	21,163,567	73.8	37,452	336
**13**	Pepper	Tulsa	19,339,367	73.3	39,840	333
**14**	Pepper	Tulsa	19,500,297	73.2	34,527	328
**15**	Pepper	Caddo	18,164,116	73.6	34,755	336
**16**	Pepper	Caddo	56,712,856	72.5	93,719	365
**17**	Pepper	Caddo	20,816,675	73.8	43,595	351
**18**	Pepper	Caddo	54,321,733	71.9	88,071	361
**19**	Pepper	Caddo	52,118,003	71.7	81,414	355
**20**	Pepper	Caddo	16,793,467	71.7	36,948	332
**21**	Pepper	Caddo	233,745,987	72.1	177,188	401
**22**	Pepper	Caddo	33,317,739	70.2	61,053	345
**23**	Pepper	Caddo	17,233,369	71.6	40,152	334
**24**	Pepper	Caddo	17,985,269	71.7	47,145	339
**25**	Pepper	Caddo	19,373,030	72.4	46,644	350
**26**	Pepper	Caddo	18,888,930	72.1	47,602	352
**27**	Pepper	Caddo	17,254,594	73.6	31,418	329
**28**	Pepper	Caddo	18,282,716	72.4	42,043	332
**29**	Pepper	Caddo	16,889,764	72.6	21,184	327
**30**	Pepper	Caddo	15,595,886	73.6	17,407	316
**31**	Pepper	Caddo	17,749,423	72.6	40,945	332
**32**	Pepper	Caddo	18,827,330	71.6	39,449	332
**33**	Pepper	Caddo	20,639,947	73.3	54,801	343
**34**	Pepper	Caddo	18,311,019	72.1	46,803	329
**35**	Pepper	Caddo	26,108,711	74.1	43,618	358
**36**	Pepper	Greer	23,694,098	73.4	55,297	372
**37**	Pepper	Cherokee	31,842,951	73.5	75,953	354
**38**	Blackberry[4]	Cherokee	27,817,549	74.2	25,337	368
**39**	Pepper	Cherokee	25,499,039	73.3	65,386	364
**40**	Pokeweed[5]	Cherokee	32,109,361	73.5	54,531	368

**41**	Pepper	Greer	42,874,718	73.8	98,452	371
**42**	Pepper	Greer	23,365,377	73.5	62,059	355
**43**	Pepper	Cherokee	25,128,347	73.7	68,147	346
**44**	Pepper	Caddo	23,799,679	74.2	54,633	360
**45**	Pepper	Caddo	22,874,040	73.7	62,086	351
**46**	Pepper	Caddo	22,114,386	73.7	58,011	355
**47**	Pepper	Caddo	21,426,816	73.8	60,260	353
**48**	Pepper	Caddo	23,756,735	73.8	62,004	345
**49**	Pepper	Caddo	19,380,648	73.4	58,563	347
**50**	Pepper	Caddo	24,657,599	73.2	45,954	359
**51**	Pepper	Caddo	23,265,636	73.3	62,594	360
**52**	Pepper	Caddo	20,305,322	73.6	52,347	356
**53**	Pepper	Cherokee	20,861,623	73.6	46,665	352
**54**	Pepper	Cherokee	22,512,401	74.1	49,587	352
**55**	Pepper	Cherokee	24,853,138	73.7	54,881	368
**56**	Pepper	Cherokee	24,066,841	74.1	59,738	360
**57**	Pepper	Cherokee	22,926,429	74.2	52,871	372
**58**	Russian thistle[6], lambs quarter[7]	Greer	20,315,789	74	31,425	316
**59**	Basil[8], Squash[9]	Cherokee	35,553,895	73.3	37,762	308
**60**	Potato[10]	Cherokee	22,519,302	73.9	46,874	351
**61**	Squash	Tulsa	23,134,039	74.1	34,016	348
**62**	Tomato	Tulsa	23,158,064	73.9	46,270	368
**63**	Squash	Cherokee	18,792,708	73.9	44,701	357

[1] NA = Samples that were analyzed throughout the study, but the initial .fastq file was corrupted when uploading the sequence data to the SRA database. [2] Pepper = *Capsicum annuum*, [3] Tomato = *Solanum lycopersicum*, [4] blackberry = *Rubus* sp., [5] Pokeweed = *Phytolacca americana*, [6] Russian thistle = *Salsola sp.*, [7] lamb's quarter = *Chenopodium sp.*, [8] Basil = *Ocimum basilicum,* [9] Squash = *Cucurbita* sp., [10] Potato = *Solanum tuberosum*

Supplementary Table S3. Mixed viral infections involving multiple viruses and virus strains detected by high-throughput sequencing (HTS).

Library ID	Virus 1	Virus 2	Virus 3
**1**	AMV	CMV	
**2**	AMV	CMV	PMMoV
**4**	AMV	CMV	PMMoV
**5**	AMV	PMMoV	
**11**	PMMoV	PYDV-CYDV	
**16**	AMV	BCTV	BCTV-PeYDV
**18**	BCTV	BCTV-PeYDV	
**21**	BWYV	CMV	
**27**	AMV	BWYV	CMV
**29**	BWYV	CMV	
**30**	BWYV	CMV	
**36**	BCTV	BCTV-PeYDV	
**46**	BCTV	BCTV-PeYDV	
**47**	BCTV	BCTV-PeYDV	
**48**	BCTV	BCTV-PeYDV	
**51**	BCTV	BCTV-PeYDV	
**54**	CMV	PYDV-CYDV	

AMV = alfalfa mosaic virus, CMV = cucumber mosaic virus, PMMoV = pepper mild mottle virus, PYDV-CYDV = potato yellow dwarf/constricta yellow dwarf virus, BCTV = beet curly top virus, BCTV-PeYDV = strain of BCTV, BWYV = beet western yellows virus

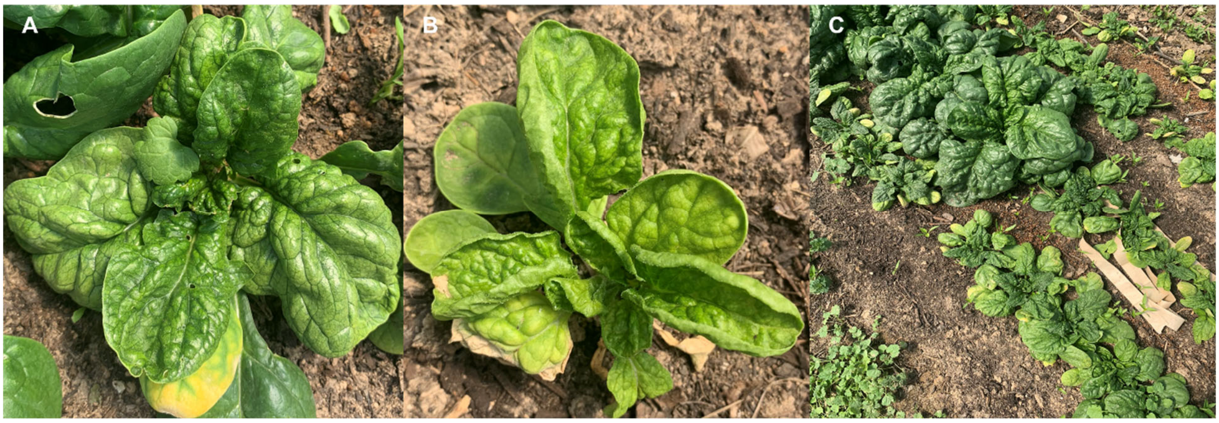

Supplementary Figure S1 CMV infection in spinach. (A) mosaic pattern and signs of early CMV infection. (B) Stunting and leathery leaves indicating more severe infection. (C) Comparison of healthy (left side) and CMV-infected spinach (right side) plants. Infection was confirmed by PCR using CMV specific primers.
